# Genomic Structural Variations Affecting Virulence During Clonal Expansion of *Pseudomonas syringae* pv. *actinidiae* Biovar 3 in Europe

**DOI:** 10.3389/fmicb.2018.00656

**Published:** 2018-04-05

**Authors:** Giuseppe Firrao, Emanuela Torelli, Cesare Polano, Patrizia Ferrante, Francesca Ferrini, Marta Martini, Simone Marcelletti, Marco Scortichini, Paolo Ermacora

**Affiliations:** ^1^Department of Agricultural, Food, Environmental and Animal Sciences, University of Udine, Udine, Italy; ^2^Istituto Nazionale Biostrutture e Biosistemi, Rome, Italy; ^3^Council for Agricultural Research and Analysis of Agricultural Economics (CREA), Research Centre for Olive, Fruit Trees and Citrus, Rome, Italy

**Keywords:** bacterial canker, genomic diversity, hypersensitivity response (HR), Illumina technology, single molecule real-time (SMRT) sequencing

## Abstract

*Pseudomonas syringae* pv. *actinidiae* (Psa) biovar 3 caused pandemic bacterial canker of *Actinidia chinensis* and *Actinidia deliciosa* since 2008. In Europe, the disease spread rapidly in the kiwifruit cultivation areas from a single introduction. In this study, we investigated the genomic diversity of Psa biovar 3 strains during the primary clonal expansion in Europe using single molecule real-time (SMRT), Illumina and Sanger sequencing technologies. We recorded evidences of frequent mobilization and loss of transposon Tn6212, large chromosome inversions, and ectopic integration of IS sequences (remarkably ISPsy31, ISPsy36, and ISPsy37). While no phenotype change associated with Tn6212 mobilization could be detected, strains CRAFRU 12.29 and CRAFRU 12.50 did not elicit the hypersensitivity response (HR) on tobacco and eggplant leaves and were limited in their growth in kiwifruit leaves due to insertion of ISPsy31 and ISPsy36 in the *hrpS* and *hrpR* genes, respectively, interrupting the *hrp* cluster. Both strains had been isolated from symptomatic plants, suggesting coexistence of variant strains with reduced virulence together with virulent strains in mixed populations. The structural differences caused by rearrangements of self-genetic elements within European and New Zealand strains were comparable in number and type to those occurring among the European strains, in contrast with the significant difference in terms of nucleotide polymorphisms. We hypothesize a relaxation, during clonal expansion, of the selection limiting the accumulation of deleterious mutations associated with genome structural variation due to transposition of mobile elements. This consideration may be relevant when evaluating strategies to be adopted for epidemics management.

## Introduction

*Pseudomonas syringae* pv. *actinidiae* (Psa) is the causal agent of bacterial canker of green-fleshed (*Actinidia deliciosa*) and yellow-fleshed kiwifruit (*Actinidia chinensis*) (Scortichini et al., 2012). The pathogen was first isolated in Japan (Takikawa et al., 1989), where the disease was reported since 1984 and, subsequently, in Italy (Scortichini, 1994) and South Korea (Koh et al., 1994). In the years 2008–2011, sudden and repeated epidemics of bacterial canker developed firstly in central Italy (Balestra et al., 2009; Ferrante and Scortichini, 2009, 2010), and, subsequently, in all the other major areas of kiwifruit cultivation such as New Zealand (Everett et al., 2011), and Chile (EPPO, 2016). In Europe, the epidemics spread to Portugal, France, Spain, Switzerland, Germany, Slovenia and Greece (Abelleira et al., 2011; Dreo et al., 2014; Cunty et al., 2015b; Holeva et al., 2015; EPPO, 2016).

Genomic and genetic analyses have soon revealed that the Psa strains causing the 2008–2011 epidemics differed significantly from those previously found in Italy (Marcelletti et al., 2011) and that the first outbreaks of kiwifruit bacterial canker in Italy (Ferrante et al., 2015) were caused by a rapid and clonal expansion of the pathogen in the cultivated areas (Marcelletti and Scortichini, 2011). Then, the availability of strains isolated in China, the area of origin of many *Actinidia* spp., and the intensive use of Illumina sequencing of bacterial genomes (Mazzaglia et al., 2012; Butler et al., 2013; McCann et al., 2013, 2017) and VNTR analysis (Cesbron et al., 2015; Ciarroni et al., 2015; Cunty et al., 2015a) paved the way to the understanding of the epidemiology of this important disease.

At present, Psa is subdivided into four biovars, three of which with distinct phylogeographic structure. Strains belonging to biovar 1 produce phaseolotoxin and have been isolated in Japan and Italy before 2008. Strains of biovar 2 produce coronatine instead of phaseolotoxin and have been isolated only in South Korea. Strains belonging to biovar 3 produce neither phaseolotoxin nor coronatine and are responsible for the global outbreak of bacterial canker of kiwifruit in recent years. Strains of biovar 5 are found only in a limited local area of Japan (Saga Prefecture), they do not produce phaseolotoxin nor coronatine and are distinct but related to biovar 2 (Fujikawa and Sawada, 2016). A fifth clade, initially identified as Psa biovar 4, has been recently described as a new pathovar, *P.s*. pv. *actinidifoliorum* (Cunty et al., 2015b; Ferrante and Scortichini, 2015). Genome analysis performed so far is consistent with the hypothesis that all Psa biovars originated from a single natural source population and established subsequent outbreaks on cultivated kiwifruit. McCann et al. (2013) highlighted the overall clonal population structure with signatures of within-pathovar, intra-biovar recombination.

Psa biovar 3 is distinct from other biovars for the virulence and the sudden world-wide epidemic spread, that has unveiled major weakness of our kiwifruit cultivation system, while calling for efforts in the clarification of its dynamics in view of future prevention. Several genome-wide diversity studies revealed that epidemics in Europe, New Zealand and Chile of Psa biovar 3 originated from independent introductions of a single founder variant from China (Mazzaglia et al., 2012; Butler et al., 2013; McCann et al., 2013) which, however, is not deemed the center of origin of the biovar 3 (McCann et al., 2017).

In this work, we examined a sampling of the Psa population that originated in Europe from the putative single introduction that occurred in 2008. Through the analysis of Illumina sequence data-sets of 11 European and one non-European Psa genomes, and through the reconstruction and comparison of two complete genomes, a picture emerged that accounts for the significant differences in the modes of genome evolution of this bacterium before and after the clonal expansion associated with the pandemic. DNA mobilization due to transposable elements was a major cause of structural differences and, at least in one case, resulted in the disruption of genes relevant in pathogen-host interaction, with an effective reduction of strain virulence on kiwifruit.

## Materials and methods

### Strains and sequencing

The strains investigated in this work and their genome data accessions are listed in Table 1.

**Table 1:** Strains and sequences used in this work.

**Strain name**	**Received as**	**Origin**	**Isolation year**	**Host plant**	**DNA sequence reference**	**HR on tobacco**	**HR on eggplant**	**Tn6212 integration**	**SRA database accession**	**GenBank accession**
CRAFRU 14.08	Psa 354	Portugal	2010	*A. deliciosa* Summer	This work	+	+	–	SRR5273023	CP019730
CRAFRU 12.29	23b	Italy (Piemonte)	2011	*A. deliciosa* Hayward	This work	–	–	–	SRR5273025	CP019732
CRAFRU 14.25	our isolate	Italy (Latium)	2012	*A. chinensis* Hort16A	This work	+	+	+	SRR5273031	n.a.
CRAFRU 12.54	1616-291a	Italy (Piemonte)	2011	*A. deliciosa* Hayward	This work	+	+	+	SRR5273030	n.a.
CRAFRU 14.10	Psa 490	Italy (Calabria)	2010	*A. chinensis* Jintao	This work	+	+	+	SRR5273029	n.a.
CRAFRU 12.64	1616-231Aa	Italy (Piemonte)	2010	*A. chinensis* Jintao	This work	+	+	+	SRR5273028	n.a.
CRAFRU 10.29	4252 A,1	Italy (Emilia Romagna)	2009	*A. chinensis* Jintao	This work	+	+	+	SRR5273027	n.a.
CRAFRU 12.50	our isolate	Italy (Campania)	2011	*A. chinensis* Jintao	This work	–	–	–	SRR5273026	n.a.
CRAFRU 14.21	37.51	France	2011	*A. chinensis* Jintao	This work	+	+	–	SRR5273024	n.a.
CRAFRU 13.27	IVIA 3729.2	Spain	2011	*A. deliciosa* Hayward	This work	+	+	–	SRR5273022	n.a.
CRAFRU 8.43	our isolate	Italy (Latium)	2008	*A. chinensis* Hort16A	Marcelletti et al., 2011	+	+	+	n.a.	AFTG00000000
CRAFRU 13.04	ICMP 18884	New Zealand	2010	*A. deliciosa* Hayward	Templeton et al., 2015	n.i.^a^	n.i.	n.i.	SRR5273021	CP011972
**ADDITIONAL SEQUENCES USED IN THIS WORK**										
7286		Italy			Mazzaglia et al., 2012				SRR364082	
ICMP 18708, V13		New Zealand			Poulter et al., unpublished^b^				n.a.	CP012179

a *Not Investigated*.

b *Deposited as Poulter, R. T. M., Poulter, G. T. M., Stockwell, P. A., Lamont, I. L., and Butler, M. I. (unpublished)*.

Genomic DNA was extracted from 1 ml of 24 h old cultures grown in Nutrient Broth with agitation using a Wizard DNA purification kit (Promega Italia, Padova, Italy) following the manufacturer's instructions. DNA was measured and checked for quality using a NanoDrop spectrophotometer (NanoDrop products, Wilmington, DE, USA). Illumina libraries were prepared as described previously (Scortichini et al., 2013) and sent to the Istituto di Genomica Applicata (Udine, Italy) for sequencing on an Illumina Genome Analyser IIx (Illumina, USA). An average of 14 million single (50 nts) reads were obtained, filtered for quality using Prinseq (Schmieder and Edwards, 2011) and further processed. The sequence reads of strain 7286, obtained by Mazzaglia et al. (2012) were downloaded from the Sequence Read Archive (SRA accession SRX105337; https://www.ncbi.nlm.nih.gov/sra). The complete genome sequence of strain ICMP ICMP 18884 (Templeton et al., 2015) and ICMP 18708 (yet unpublished but made available by Poulter, R. T. M., Poulter, G. T. M., Stockwell, P. A., Lamont, I. L. and Butler, M. I.) were obtained from the NCBI nucleotide database and used as comparative reference for non-European strain.

Genomic DNA extracted from strains CRAFRU 12.29 and CRAFRU 14.08 was also sent for single molecule real-time (SMRT) sequencing to the University of Washington PacBio Sequencing Services. The genomes were then finished with Sanger sequencing using a primer walking approach on PCR fragments amplified from putatively adjacent contig ends, as resulted by scaffolding using ICMP 18708 as a reference; fragments were sent for sequencing to Genelab, Casaccia, Italy. Sequences were edited and manipulated using Seaview (Gouy et al., 2010) and Ugene (Okonechnikov et al., 2012).

### Sequence analysis

Preliminary read alignments and alignment manipulation were carried out using widely used tools such as BWA 0.5.5 (Li and Durbin, 2009), SAMtools 0.1.16 (Li et al., 2009) and PICARD tools (http://picard.sourceforge.net). SNP calling was carried out with the GATK package (McKenna et al., 2010); SNPs call was supported by a depth of coverage of at least 5 and a consensus of at least 95% of the aligned reads. Briefly, reads of each strain were preprocessed for quality using SGA [1], aligned on either CRAFRU 14.08 or ICMP 18884 chromosomes using BWA, indexed, sorted and reformatted using SAMtools, organized into reads-groups by PICARD tools; the resulting sam file was processed by GATK, and the output VCFs read by a bash script that checked each SNP for support and organized the results in a table for manual examination. Tablet (Milne et al., 2010) was used for the visualization of the alignments.

The genome assemblies for SMRT sequencing data were first generated using the hierarchical genome-assembly process (HGAP) [2], then Illumina reads were mapped and searched as described above for SNPs to identify incongruences; the alignments were then inspected with Tablet. For the genome of CRAFRU 14.08, the 4 gaps remaining were closed by Sanger sequencing using a primer walking approach. Once completed, the genomes were aligned with Mauve (Darling et al., 2004) and MUMmer (Delcher et al., 2002). The insertion thus identified were exported and annotated using Blast, the ISFinder database (Siguier et al., 2006), and the annotation service of Insertion Sequences (IS) provided by the ISsaga (Insertion Sequence semi-automatic genome annotation) engine (Varani et al., 2011).

Reads of several strains were aligned to the complete genome as described and visualized with IGV (Robinson et al., 2011), allowing the visual comparison of mapped reads densities. On the basis of the information gained by visual inspection, specific regions were selected for targeted assembly, that was carried out with Mapsembler2 ver. 2.2.4 (Peterlongo and Chikhi, 2011).

For reference based assemblies of relatively small DNA regions such as PAC_ICE2, and for the assembly of unmapped reads for gene discovery, the Illumina reads of the 12 European strains were processed with Edena (Hernandez et al., 2008). From the same datasets, full genomes were drafted with SPAdes (Bankevich et al., 2012) and scaffolded with Ragout (Kolmogorov et al., 2014) having the complete genome of CRAFRU 14.08 as a reference. Using Mauve and MUMmer the drafts were aligned to complete genomes, visualized and the polymorphic regions exported. Mobile elements and repeats were identified with Juxtaposer [3], the ISsaga engine, and the Tandem Repeats Finder program (http://tandem.bu.edu).

Structural variations were also searched using the split-read, (i.e., chimeric read) approach [4] with the aid of the program bbduck of the suite BBMap (*Bushnell* B. - sourceforge.net/projects/bbmap/).

The above listed tools were integrated with several *ad-hoc* Perl scripts into Bash scripts and run on Linux instances launched on the infrastructures of the DIAG (http://www.igs.umaryland.edu/resources/irc/) and CyVerse (Merchant et al., 2016) projects.

### Plantlet inoculations

To investigate whether or not Psa strains were impaired in their within plant colonization capabilities, micropropagated kiwifruit plantlets *A. chinensis* (cv. Soreli) at the stage of 6 leaves, provided by Azienda Agricola Fanna Giampaolo (Moimacco, Italy) were used for plantlet inoculation. Bacterial strains grown for 24 h in Nutrient Broth with agitation were washed twice and resuspended in 0.9% saline solution in concentration of 1–2 × 10^9^ CFU/ml. Plantlets were cut from callus, dipped in the inoculum and transferred to a fresh medium. Control plants were dipped in sterile saline. Plantlets were inoculated in five repetitions per experiment. After 10 days the plantlets were collected, cut into two halves (about 3 cm from inoculation point), and DNA was extracted from each subsample according to standard protocols (Doyle and Doyle, 1990). The bacterial populations were quantified by qPCR according to published protocol (Gallelli et al., 2014). For statistical analysis, carried out with R (R Core Team, 2013), the median of three PCR reactions was used.

### Leaf inoculations

To compare the capability of strains to induce disease symptoms and to determine their growth *in planta, Actinidia chinensis* (cv. Dorì®) leaves were inoculated with the method described previously (Marcelletti et al., 2011). Leaf areas of approximately 1 cm in diameter were inoculated at the concentrations of 1–2 × 10^6^ CFU/ml. For each experiment, 10 leaves were inoculated in four sites. Control plants were treated using solely sterile 0.85% NaCl. Two, 6, 15, and 22 days after inoculation, 10 leaf disks of about 0.5 cm of diameter were sampled and ground in 1 ml of sterile saline, then serial ten-fold dilutions were counted by colony growth onto nutrient agar supplemented with 3% of sucrose (NSA).

Hypersensitive responses were tested by infiltrating aqueous bacterial suspensions at 1–2 × 10^8^ CFU/ml on fully expanded tobacco and eggplant leaves using a needless syringe. The development of typical hypersensitivity response was checked within 48 h after infiltration. Assays were repeated three times.

### Other wet lab methods

To determine the excised/integrated state of Tn6212, primers (Table S1) were designed on the inner and outer borders of the transposon. PCRs with primer pairs fX1/rX2; fX1/rX4, and fX3/rX4 were performed with the automated One Advanced thermocycler (EuroClone, Celbio, Milan, Italy) in 25 μl reactions containing 200 μM of each of the four dNTPs, 0.4 μM of each primer, 1.5 mM MgCl2, 0.625 units of GoTaq Flexi DNA Polymerase (Promega, Madison, WI, USA) and 1 μl of diluted bacterial DNA (5 ng/μl). The PCR program consisted of initial denaturation for 2 min at 94°C; 35 cycles of 1 min at 94°C, 45 s at 58°C, 1 min at 72°C; and a final extension for 8 min at 72°C.

PCR products were separated by electrophoresis in a 1% agarose gel, stained with ethidium bromide, and captured with a DigiDoc-It imaging system (UVP, Cambridge, United Kingdom).

## Results and discussion

### Differential hypersensitive response (HR) of Psa CRAFRU 12.29 and CRAFRU 12.50 is due to insertional inactivation of the *hrp* gene cluster

**Figure 1:**
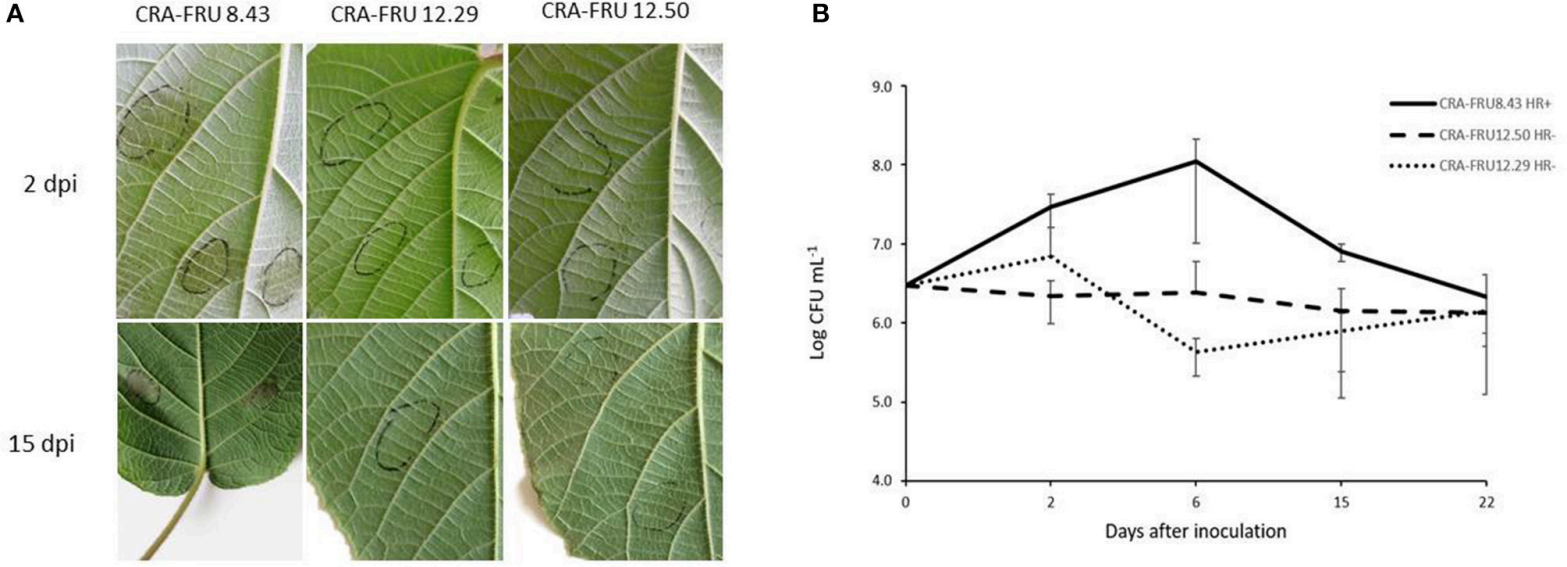
**(A)** Symptoms on kiwifruit leaves 2 and 15 days post inoculation (dpi) with CRA-FRU 8.43, CRA-FRU 12.29, and CRA-FRU 12.50. **(B)** Population dynamics of Psa strains CRAFRU 8.43 (HR+), CRAFRU 12.29 (HR–), and CRAFRU12.50 (HR–) after inoculation of kiwifruit leaves.

Psa biovar 3 strains isolated in different regions of Europe were investigated to assess their phytopathogenic and genomic diversity. While most strains, as expected, induced HR in eggplant and tobacco leaves when infiltrated at concentrations of 1–2 × 10^8^ CFU/ml, strains CRAFRU 12.50 and CRAFRU 12.29 failed in eliciting HR (not shown). Strains CRAFRU 12.50 and CRAFRU 12.29 were also compared with the reference strain CRAFRU 8.43 for their ability to colonize *A. chinensis* leaves. Visual observations clearly revealed differences between CRAFRU 8.43 (HR+), that caused leaf spots, on one hand, and CRAFRU 12.29 (HR–) and CRAFRU 12.50 (HR–), on the other, that failed in inciting foliar symptoms. The estimate of bacterial concentration in leaves in the 22 days after inoculation, reported in Figure 1, showed that the population sizes of strain CRAFRU 12.29 and CRAFRU 12.50 did not increase during the assay time, while those of the virulent strain CRAFRU 8.43 peaked up to 100 times the inoculum. Thus, although the bacterial populations of HR– strains did not increase as much as the wild type, the bacteria remained detectable after 22 days. Further experiments carried out on micropropagated plantlets inoculated by dipping, revealed that the CRAFRU 12.29 cells move within the stem and were detectable by PCR in the stem segments above the point of inoculation 10 days after the dipping (results not shown).

In a preliminary SNPs analysis, based on Illumina sequencing data, only one nucleotide difference could be detected between the HR– strain CRAFRU 12.29 and the HR+ strain CRAFRU 14.08.

**Figure 2:**
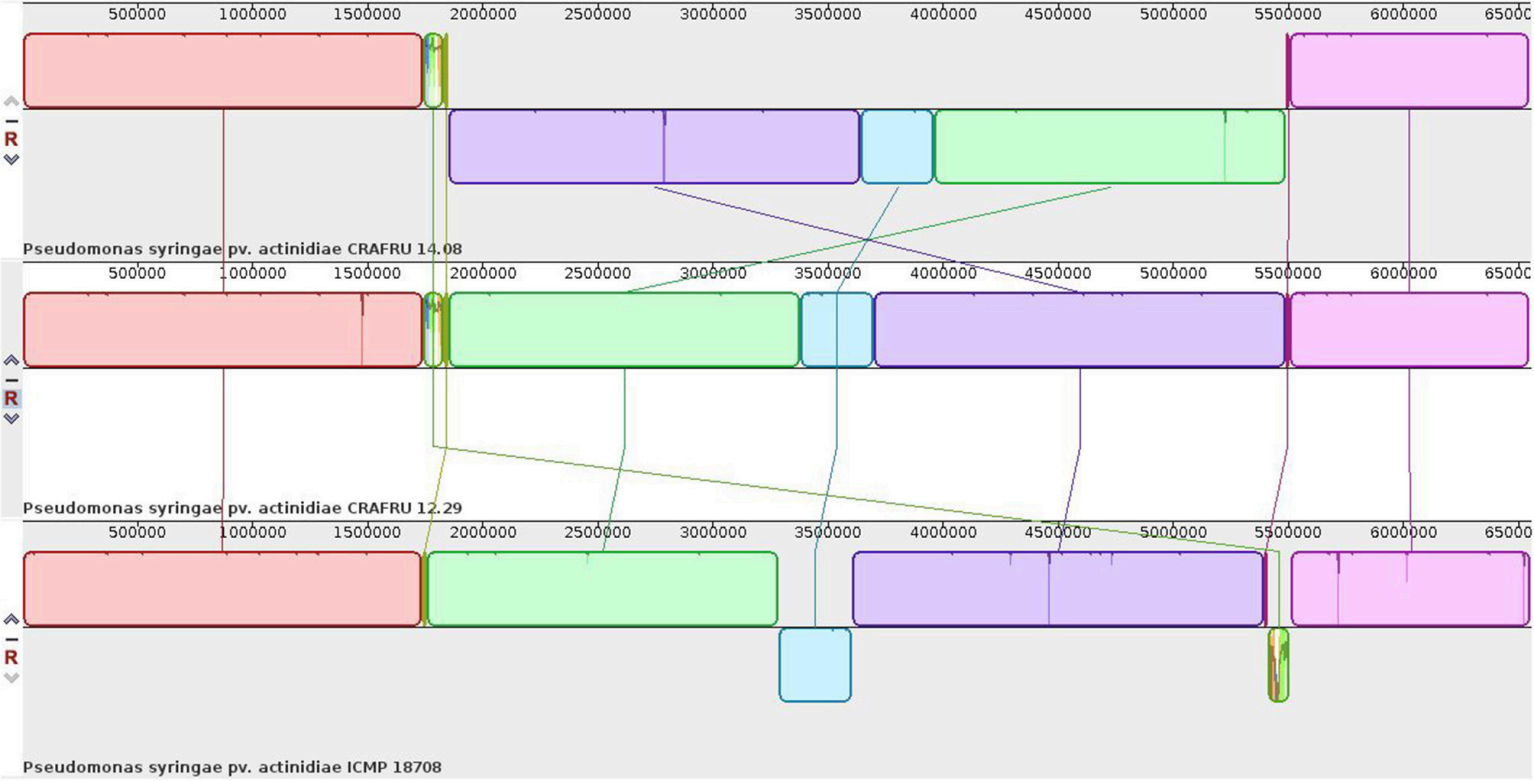
Mauve alignment of the chromosomes of strains CRAFRU 14.08, CRAFRU 12.29, and ICMP 18708.

Hence, the genome sequences of strains CRAFRU 14.08 and CRAFRU 12.29 were completed by SMRT (Single Molecule, Real Time) and Sanger sequencing. The resulting finished chromosomes, as shown in the alignment of Figure 2, differ for several structural features.

First of all, CRAFRU 14.08 displays a large inversion of about half of the chromosome (3,637,997 nts) as compared to CRAFRU 12.29. The inversion occurred by recombination of the two identical copies of the gene encoding an integrating conjugative element protein of the PFL_4705 family, that are located, together with some other complete and incomplete copies, at position 1850000–1858000 and 5488000–5500000 in the chromosome of CRAFRU 12.29. Chromosome inversions have been reported to affect gene expression and occasionally the phenotype (Cui et al., 2012). However, whether or not the large genome inversion in CRAFRU 14.08 is associated with phenotype could not be determined in the present study.

**Figure 3:**
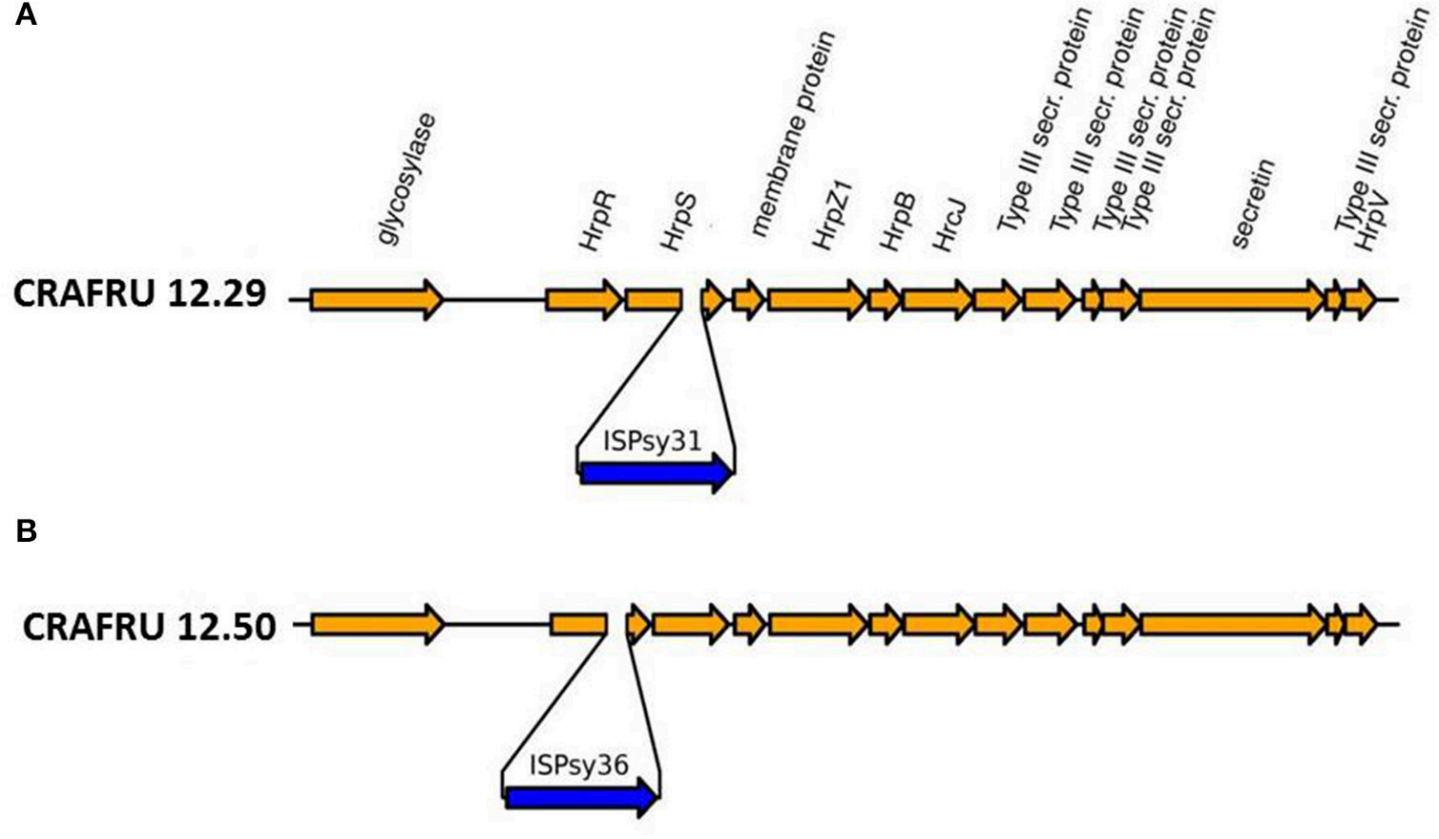
Drawing of part of the *hrp* cluster of Psa, with the location of the insertion of **(A)** ISPsy31 in strain CRAFRU 12.29 and **(B)** ISPsy36 in strain CRAFRU 12.50.

The second major difference in strain CRAFRU 12.29 concerns a 1,700 bp integrative sequence, encoding an integrase and an IS3/IS911 transposase. This small integrative unit was inserted in the *hrpS* gene, within a transcriptional unit that spans several components of the type III secretion system, including the gene encoding harpin, *hrpZ* (Figure 3). Since, according to annotation and Blast searches, there are no other copies of *hrpZ* in the genome of Psa CRAFRU 12.29, the lack of expression of *hrpZ* may conceivably be the reason for the reduced virulence on kiwifruit and inability to elicit HR on eggplant and tobacco leaves. The phenotype is indeed reminiscent of previously characterized *hrpZ* deletion mutants (He et al., 1993).

The mobilization of IS3/IS911 elements has been already reported by Butler et al. (2013), who found that in the comparison of Pac_ICE1 from four New Zealand strains (ICMP 18708, ICMP18800, TP1, and 6.1) the presence of an IS element of the type IS3/IS911 in strain 6.1 was the only difference. They designated this small transposable element ISPsy31 at the IS Finder database (Siguier et al., 2006) and we will follow this nomenclature. As remarked by Butler et al. (2013), ISPsy31 is predicted to have two, partially overlapping reading frames associated with a 21 frame shift (the typical pattern found in IS3/IS911 type elements). Hence, although ISPsy31 encodes no functions other than those involved in its mobility, it may significantly impact the behavior of the pathogen in its interaction with the host.

There are many copies of ISPsy31 in the Psa genome. In strain CRAFRU 12.29 we counted 52 complete and five incomplete copies in the chromosome, and two complete copies in the plasmid. With the notable exception of the one interrupting *hrpS*, all other ISPsy31 copies are in corresponding positions in the chromosomes of strains CRAFRU 12.29 and CRAFRU 14.08.

On the other hand, strain CRAFRU 14.08 genome displays (position 5223542–5224799) the insertion of another IS element of the IS3/IS911 family, related to but well distinct from ISPsy31, and designated as ISPsy37 at the ISFinder database (Siguier et al., 2006). There are two copies of this transposon in CRAFRU 14.08, and only a single occurrence in CRAFRU 12.29.

Finally, one variation associated with variable number tandem repeats (VNTR) was also scored at positions 2787533–2786633 in CRAFRU 14.08, in additions to two unique SNPs (see below). Differences between the chromosomes of CRAFRU 14.08 and CRAFRU 12.29 are summarized in Table S2.

The finding that a small transposon insertion caused the loss of the ability to elicit the HR response in CRAFRU 12.29 prompted us to investigate whether or not the phenotype of the second HR– strain, CRAFRU 12.50, was associated to the same genomic event. To this end, we comparatively analyzed the ILLUMINA data-sets for discrepancies in reads coverage between the HR– and HR+ strains. Read coverage for the genome of the HR- strain CRAFRU 12.50 was similar to all HR+ strains in the locus of insertion of ISPsy31 in CRAFRU 12.29, but was markedly different from any other genome in a locus 1,273 nts upstream (Figure S1). Sorting the CRAFRU 12.50 reads using Mapsembler (Peterlongo and Chikhi, 2011) with sequence starters located on both sides of this locus (corresponding to position 1,473,520 in CRAFRU) we could extend the left side starter by 24 nts upstream and the right starter by 25 nts downstream, revealing the boundaries of a transposon tentatively identified as a copy of ISPsy36, inserted in the *hrpR* gene (Figure 3B).

### Structural diversity in the chromosomes of the European population of Psa biovar 3

The availability of finished genomes of European Psa strains allowed to precisely map SNPs in additional 10 genomes (Table 1) of strains isolated in Europe, using Illumina data, as summarized in Table 2 (to help following the results in this and the following sections, a tree based on distance among genomes of the Psa strains as deduced from SNPs and including all strains mentioned in this work has been included as Figure S2). According to SNP analysis, a single difference between the chromosomes of strains CRAFRU 14.08 and CRAFRU 12.29 was scored, at position 39328331 in CRAFRU 14.08. Comparison of the two finished chromosome sequences using MUMmer (Delcher et al., 2002) revealed an additional SNP at position 2736260; that position corresponds to a transposase gene that is present in several copies in the genome and therefore was not detectable by read mapping (Table S2). In summary, the SNP comparison of the 12 European Psa genomes revealed that they differ from each other in 0 to 8 sites, on a total of 19 polymorphisms detected.

**Table 2:** SNPs identified among the strains used in this work by Illumina reads mapping. Position relative to the chromosome of CRAFRU 14.08.

**Strain Position**	**CRAFRU 12.64**	**CRAFRU 10.29**	**CRAFRU 12.50**	**CRAFRU 12.29**	**CRAFRU 14.21**	**CRAFRU 14.08**	**#7286**	**CRAFRU 13.27**	**CRAFRU 8.43**	**CRAFRU 14.25**	**CRAFRU 12.54**	**CRAFRU 14.10**
32022	G	G	G	G	G	G	G	G	C	G	G	G
1537885	C	T	C	C	C	C	C	C	C	T	C	C
1791521	G	G	G	G	G	G	C	G	G	G	G	G
1791522	G	G	G	G	G	G	C	G	G	G	G	G
2109838	C	C	C	C	C	C	C	C	T	C	C	C
2554115	G	G	A	G	G	G	G	G	G	G	G	G
3540152	A	T	A	A	A	A	A	A	A	A	A	A
3540154	C	T	C	C	C	C	C	C	C	C	C	C
3932833	C	C	C	T	C	C	C	C	C	C	C	C
4207959	C	C	C	C	C	C	C	C	C	C	T	C
4262863	G	G	T	G	G	G	G	G	G	G	G	G
5267844	C	A	C	C	C	C	C	C	C	C	C	C
5268734	C	C	C	C	C	C	A	C	C	C	C	C
5346399	A	T	T	T	T	T	T	T	T	T	T	T
5379834	C	C	C	C	A	C	C	C	C	C	C	C
5719829	G	G	G	G	G	G	G	G	T	G	G	G
5803673	C	C	C	C	C	C	G	C	C	C	C	C
6189845	C	C	T	C	C	C	C	C	C	C	C	C
6357274	C	C	T	C	C	C	C	C	C	C	C	C

The SNP analysis reported here supports the assertion of Butler et al. (2013) that the clonal populations in New Zealand and Chile are undergoing divergence, but as yet the frequency of idiosyncratic SNPs is less than one per Mb. A similar rate was determined in this work for European strains, as it was also anticipated by Mazzaglia et al. (2012). However, these figures are significantly lower than those reported by McCann et al. (2013) who identified 28–70 polymorphisms among the four Italian strains included in their study. The explanation of this inconsistency may lay in the fact that for three out of the four strains compared by those Authors, they used the data from *de novo* draft assemblies deposited in the database by Marcelletti (Marcelletti et al., 2011), Butler (Butler et al., 2013), and Mazzaglia (Mazzaglia et al., 2012), respectively, and *de novo* assembly is much more error prone than the conservative read mapping method used in this work (Trivedi et al., 2014).

Mazzaglia et al. (2012) identified the presence, in the chromosome of Psa, of a divergent genomic island ~100 kb long, similar to PPHGI-1, an integrative conjugative elements (ICE) described earlier in *P. syringae* pv. *phaseolicola* (Pitman et al., 2005), and also similar to PsyrGI-6, an ICE of *P. syringae* pv. *syringae* B728a (Feil et al., 2005). The genomic island was analyzed in more detail by Butler et al. (2013), who named Pac_ICE2 the type shared by European strains of Psa (in contrast with Pac_ICE1, for New Zealand strains, Pac_ICE3, for Chilean).

Butler et al. (2013) reported that the islands in ICMP 18708 (New Zealand), ICMP 18744 (Italy) and ICMP 19455 (Chile) were broadly syntenic, although the sequences shared by the ICEs were significantly divergent (~85% identical). Two regions with high conservation were detected, corresponding to transposons named Tn6211 and Tn6212. While Tn6211 occupy distinct positions in each of the three ICE types, the second conserved region (bases 55201–71516 in Pac_ICE1 from ICMP 18708), designated Tn6212 and almost identical in all ICEs, was syntenic in the three ICE types.

**Figure 4:**
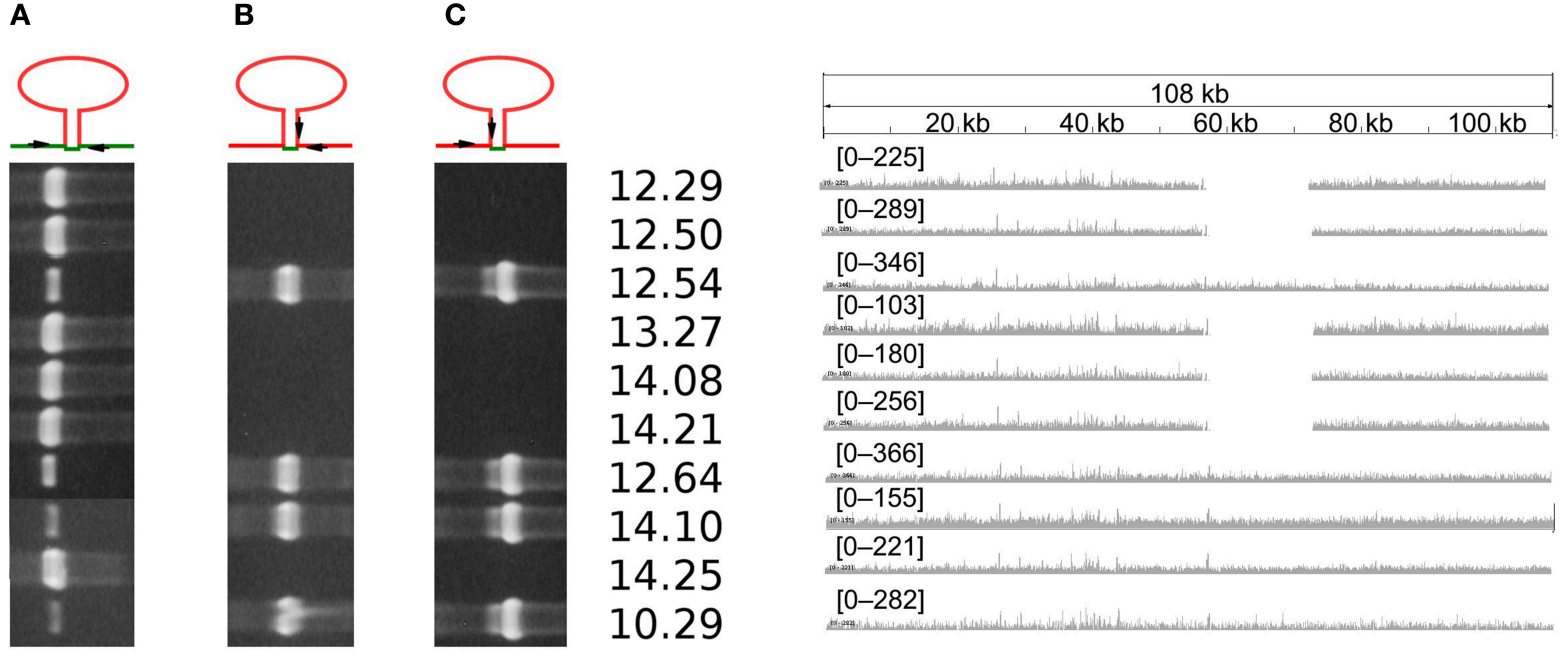
Evidence of integration/excision of Tn6212. **(Left)** Agarose gels of PCR amplification products with (A) primers fX1/rX4 (686 bp) that amplify the chromosome region resulting from excision, (B) primers fX3/rX4 (739 bp) that amplify the downstream transposon junction, and (C) primers fX1/rX2 (933 bp) that amplify the upstream transposon junction, as indicated in the top scheme of PCR primers positions. **(Right)** Density of reads mapping on Tn6212 and flanking regions. The numbers indicate the CRAFRU strains.

Mapping of Illumina reads examined in this work revealed two distinct types of Pac_ICE2 among the 12 European Psa genomes. The Illumina reads from five strains (namely CRAFRU 12.50, CRAFRU 12.29, CRAFRU 14.21, CRAFRU 14.08, and CRAFRU 13.27) did not cover the about 16.3 kbp of Tn6212 (Figure 4). “Split reads” containing Tn6212 flanking sequences were also found suggesting that the transposon was excised.

PCR carried out with primers placed on the borders of Tn6212 (Figure 4) provided confirmation of the excision and loss of Tn6212 in the named five strains: with their DNA extracts as templates, both the PCRs with primers located on left end of Tn6212 and flanking region, and the PCRs with primers located on right end of Tn6212 and flanking region, failed to amplify a DNA fragment of the expected size. Conversely, PCRs with primers specific for the left and right flanking regions amplified a DNA fragment that was 686 bp in length, i.e., lacking the Tn6212 sequence. Unexpectedly, the DNA samples from the other strains were positive not only to PCRs designed to amplify the ends of Tn6212 and flanking regions, but also primed amplification of the 686 bp DNA fragment with primers specific for the left and right flanking regions.

Since the DNA samples were prepared from 24 h old liquid cultures started from single colonies, we hypothesize that Tn6212 may occur with high frequency *in vitro*, so that at the time of DNA extraction the sample contained a mixture of genomes with and without Tn6212 integration. A similar hypothesis may explain the incongruity of the results concerning strain CRAFRU 14.25, that showed reads coverage of the Tn6212 region but no amplification products with primers located on its ends. Since the sequencing was carried out more than 1 year before PCRs, we hypothesize that subculturing ultimately selected genomes missing Tn6212.

The evidence of optional and frequent excision of Tn6212 raised the question of its potential role in the interaction with the plant host, that could warrant its maintenance in the pathogen population over time and its detection in fresh strains.

Tn6212 has been reported to be the Psa specific part that distinguished ICEs of Psa and *Ps. syringae* pv. *phaseolicola* (Psp). McCann et al. (2013) pointed out the presence within the Tn6212 region of genes that may be implicated in the interaction with the plant host, such as those encoding a predicted enolase and various transporters, including an ortholog of DctT (a putative di- carboxylic acid transporter with N-terminus predicted to be targeted to the Type III Secretion System) and a methyl-accepting chemotaxis protein predicted to be involved in taxis toward malate.

**Figure 5:**
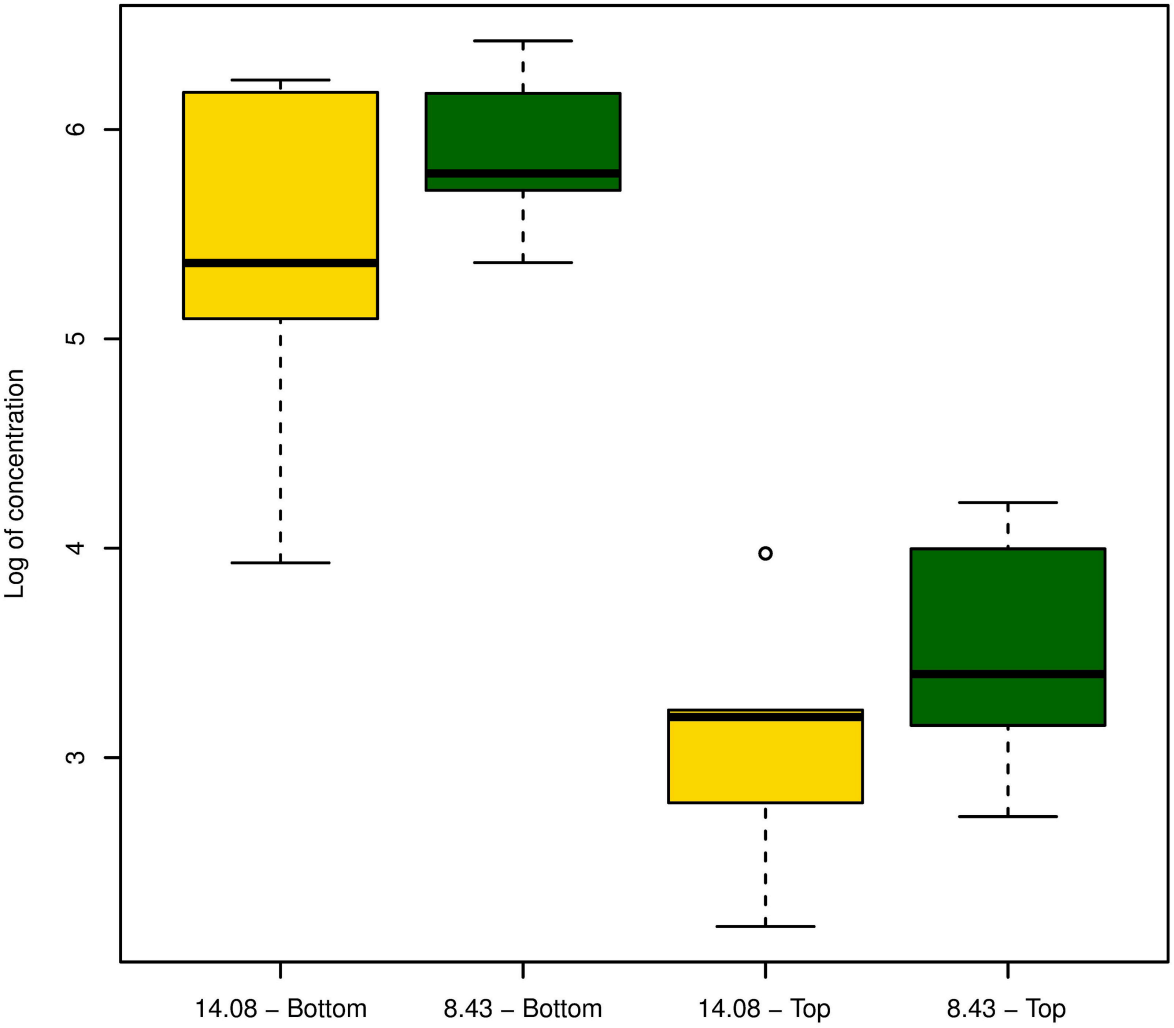
Boxplot of the estimated bacterial population in the upper **(Top)** and lower **(Bottom)** part of the stem 10 days after inoculation with strains CRAFRU 8.43 and CRAFRU 14.08.

In an attempt to detect differences in virulence and within-plant colonization of strains, we inoculated plantlets with strain CRAFRU 8.43 and CRAFRU 14.08 and, after 10 days incubation, estimated by qPCR the bacterial population in the point of inoculation (“bottom” in Figure 5) and in the stem segment 3 cm above (“Top” in Figure 5). Although the bacterial cell number estimated of CRAFRU 8.43 were higher, the detected difference was not statistically significant.

The optional excision of Tn6212 is the only significant variation in ICE2 among the 12 genomes examined. In fact, ICE2 resulted identical in all strains except for a single polymorphism in strain CRAFRU 10.29 at position 51525.

Furthermore, we examined the results of Illumina re-sequencing of all Psa strains with the aim of discovering new genes possibly acquired during clonal expansion. Following reads mapping on the complete genome of strain CRAFRU 12.29, we selected and assembled the Illumina reads that were not mapped. After filtering for Tn6212 (missing in the reference) sequences, we obtained in total 175 contigs for a total of 105,000 nts. The encoded amino acids sequences whose function could be recognized according to RAST annotation were exclusively phage associated proteins (Table S4). Hence, we could find no evidence of gene gain in our sample of 12 European genomes, reveling a picture divergent from that described by Colombi et al. (2017) who showed the acquisition by strains isolated in New Zealand of exogenous integrative conjugative elements carrying copper resistance genes during clonal expansion.

To confirm that genome diversity in the European strains is mostly due to rearrangement of self-genetic elements, the Illumina dataset was used to investigate structural changes in the chromosomes of the collections of 10 European strains, with different approaches.

We mapped the Illumina reads from all strains on both the CRAFRU 12.29 and CRAFRU 14.08 chromosomes and visualized the alignments in the regions covering the structural changes that differentiate those chromosomes among themselves. As a result, we found that the ISPsy31 insertion in CRAFRU 12.29, as well as the ISPsy37 insertion and the large inversion in CRAFRU 14.08, and the ISPsy36 insertion in CRAFRU 12.50 were unique in the respective strain chromosomes and not shared by any other of the remaining European strains. We therefore focused on the detection of specific structural changes in the chromosomes of the other strains.

To this end, we prepared an inventory of the mobile elements that can be detected in the two complete chromosomes of European Psa, CRAFRU 12.29 and CRAFRU 14.08 (Figure S3), then mapped their ends on the assemblies of other strains to detect traces of transposon mobilization. By using this approach, we found contigs ending with sequences associated with mobile element borders that were not present in the reference chromosome. In particular, we found IS3 related sequences in unique positions in CRAFRU 12.64 and CRA 8.43, and an IS3 related sequence present in the same position in both CRAFRU 13.27 and CRAFRU 10.29.

The assemblies were also scaffolded using CRAFRU 12.29 genome as a reference and visualized, allowing the detection of an inversion around position 5508000 (CRAFRU 12.29 numbering) in strain CRAFRU 8.43.

### Comparison of chromosomes of European vs. New Zealand Psa biovar 3 strains

The comparison of the European strain CRAFRU 12.29 and the two complete genomes of New Zealand strains that were available from NCBI in October 2016, i.e., strains ICMP 18708 and ICMP 18884, showed substantial synteny of the chromosomes (Figure 2).

As previously noticed the sequences diverged largely in the ICE region, while divergence was much less in the rest of the genome. As it has already been reported for other strains (Butler et al., 2013) the ICE is inserted in a different lysine tRNA site in the genomes of European Psa strain CRAFRU 12.29 and in the New Zealand strain ICMP 18708/18884.

Excluding the ICE region, the chromosomes of the two New Zealand strains were identical to each other except for seven SNPs (including single nucleotide indels), according to the results of direct comparison using MUMmer (Delcher et al., 2002) and Mauve (Darling et al., 2004). Two of the indels occurred in homopolymer stretches and were not confirmed by our Illumina sequencing and reads mapping of strain ICMP 18884. Thus, the number of single nucleotide variations between the two New Zealand strains were similar to that occurring among the European strains. Conversely, 27 SNPs (including indels) and three sequence variations affecting multiple nucleotides were detected between the European Psa strain CRAFRU 12.29 and the New Zealand strain ICMP 18884 in the remaining (after exclusion of ICE) about 6 Mb of the chromosome (pos 1–5410820 and 5511674–6555571, strain ICMP 18708 numbering). This finding is in substantial agreement with the hypothesis that Psa strains originating the epidemics in Chile, New Zealand and Europe were independently invaded by Pac_ICE1/3, supporting the notion that this ICE may contain genetic elements that significantly affect the virulence of the pathogen.

In addition to SNPs, several genome rearrangement events distinguished the genome of the European Psa strain CRAFRU 12.29 and the New Zealand strains ICMP 18708/18884, as presented in Table S3. Major events include the insertion of a copy of a mobile selfish genetic element of the group named bacterial group II intron reverse transcriptase/maturase in CRAFRU 12.29 at positions 1023375–1025252. Proteins in this group have an N-terminal reverse transcriptase (RNA-directed DNA polymerase) domain (pfam00078) followed by an RNA-binding maturase domain (pfam08388). This mobile element is present in 14 copies in CRAFRU 12.29 and 13 copies in ICMP 18708/18884 genomes.

On the other hand, ICMP 18708 and ICMP 18884 are characterized by a similar event, the insertion of another distinct bacterial group II intron reverse transcriptase/maturase starting at position 5715260 and ending at position 5717133. Also this transcriptase/maturase is present in several identical copies in the Psa genomes, namely 14 copies in ICMP 18708 and 13 copies in CRAFRU 12.29, respectively. There are, in total, 54 protein annotated as bacterial group II intron reverse transcriptase/maturase in each of the two genomes in comparison. Another major difference between the two genomes concerns an insertion of two transposase genes at positions 3287490–3288700 in a DNA region that includes sequences encoding IS630 transposases, a phage invertase and related proteins that are associated with a 316 kb inversion in ICMP 18708/18884. Another IS630 insertion that is specific of ICMP 18708/18884 occurs in those genomes at position 6522179–6523356 (ICMP 18708 numbering). In ICMP 18708/18884 there are 61 complete and five incomplete IS630 transposases, while CRAFRU 12.29 displayed 59 complete and five incomplete copies of this gene. Two minor variations associated with repeats of variable lengths were also scored, one of which corresponding to the same repeat region that differentiated CRAFRU 14.08 from CRAFRU 12.29.

## Conclusions

Mobile DNA elements contribute to bacterial evolution, as their ability to mobilize themselves and unrelated DNA in their proximity can lead to genome rearrangements that affect the microorganism phenotype (Bardaji et al., 2011). Their role in improving fitness and, potentially, pathogenicity and virulence of phytopathogenic bacteria is well established (Jackson et al., 2011). Many studies stressed the role of mobile DNA dependent gene gain in pathogen populations during epidemics, leading to the differentiation and development of more adapted clones (Holden et al., 2009; Mutreja et al., 2011; Santagati et al., 2012; Petrovska et al., 2016). Psa biovar 3 represents a relevant example of such a process, considering the primary role of mobile DNA mediated horizontal genetic transfer (particularly the gain of ICE) in its emergence as a pandemic pathogen of kiwifruit, according to several studies (Marcelletti et al., 2011; Butler et al., 2013; McCann et al., 2013, 2017).

However, Mobile DNA-induced mutations are often deleterious (Wu et al., 2015), and transposable elements have been regarded as a sort of genomic disease (Wagner, 2009). Loss of fitness due to the accumulation of deleterious mutations has been reported for small, obligate asexual populations, as these are incapable of reconstituting highly fit genotypes by recombination or back mutation (Lynch et al., 1993; Moran, 1996).

According to the results of a pangenomic study by Bolotin and Hershberg (2015), while non-clonal species diversify through a combination of changes to gene sequences (gene loss and gene gain), gene loss completely dominates as a source of genetic variation among clonal species, for which it needs to be taken into account as a potential dominant source of phenotypic variation. In the case of Psa biovar 3, we report here a relevant number (considering the small sample) of transposon mediated structural variations, occasionally impairing relevant phenotypic aspects of the interaction with the host, as occurred in the genome of strains CRAFRU 12.29 and CRAFRU 12.50 where a ISPsy31 insertion in the *hrpS* gene and a ISPsy36 insertion in the *hrpR* gene, respectively, disrupted the functionality of the TTSS. In all cases, structural variations implied rearrangement of self-genetic elements and not incorporation of external DNA.

There is a growing body of evidence supporting the hypothesis of two phases in the recent evolution of Psa biovar 3, with a landmark in the initiation of the worldwide pandemic in 2008. The SNP based comparisons (this work, McCann et al., 2017), as well as the evidence of independent invasions of ICE (Butler et al., 2013), suggest the preservation of within biovar diversity in the natural environment of the region of origin and during initial spread in China, before pandemic initiation. In this phase, acquisition of exogenous DNA through mobile DNA and selection for increased fitness were drivers of the evolution, promoting the emergence of adapted individuals. Also in this phase, recombination (intra- and inter-pathovar; McCann et al., 2013, 2017) and selection limited the proliferation of transposons and the deleterious mutations associated to DNA mobilization.

A new phase began with the introduction of adapted highly virulent strains from China into the kiwifruit cultivated areas in Europe, Chile and New Zealand. In Europe, Psa biovar 3 established and spread clonally in an ecological niche lacking competitive selection, such as that represented by the highly sensitive *A. chinensis* cv. Hort 16A. The results of this study show that the new phase was associated to an increase in the number of small transposons in the bacterial genome, with rearrangements leading to gene loss rather than to gain of functions by horizontal transfer. The data collected herein would suggest that clonal spread of the pathogen in a free ecological niche occurred with no access to the environmental gene pool, with diversification through rearrangement of self-genetic elements, and in the absence of the recombination-selection process that mitigates genome degeneration associated with transposon mobilization (Bast et al., 2016).

This suggestion is corroborated by the genome comparisons between European and New Zealand strain. According to the SNPs analysis presented in this and other papers (Mazzaglia et al., 2012; Butler et al., 2013), SNPs differences between the two geographically distinct groups of strains are one order of magnitude larger than within group SNPs differences, supporting the notion that the separation the European and New Zealand strains consistently predates the initiation of clonal expansion in Europe; conversely the mobile DNA associated structural differences are not larger between geographically distinct groups than within groups. This discrepancy, and the isolation of variant strains defective in virulence, are consistent with the view that the clonal expansion in the open niche of cultivated kiwifruit would be associated with genomic diversification through structural rearrangement with relaxation of the natural selection pressure against deleterious traits. This issue may be relevant for our understanding and management of epidemics.

Evidence of gene gain associated with the emergence of copper-resistant strains was recently reported by Colombi et al. (2017) for Psa in New Zealand, while we found no gene gain by European strains, variant strains resulting from rearrangement of self-genetic elements. The different outcomes of the surveys may be related with differences in the environmental conditions, epidemic dynamics or disease management, such as timing of the disease spread on the territory, introduction of tolerant cultivars, use of containment measures directed to the reduction of the inoculum size (particularly copper treatments) or to the reduction of pathogen dispersal and the establishment of conducive conditions for the epidemics (pruning, girdling, cultivation under cover), prevalence of the crop in the region (Vanneste, 2017).

Modern strategies for the management of destructive epidemics, such as that caused by Psa biovar 3 on kiwifruit, may benefit from the awareness of their effect on short-term genome evolution and population structure of the pathogen. The results presented in this paper would suggest that strategies that do not promote recombination and preserve the clonal structure of the invasive microorganism may be associated with lower risk of developing variant strains with enhanced fitness or virulence.

## Author contributions

GF, MS, and PE conceived the work, designed the experiments and wrote the paper. ET made the libraries. PF performed HR assays and leaf inoculations. FF carried out plantlets inoculation and quantitative PCRs. MM performed other wet lab methods. GF, CP, and SM analyzed the sequence data.

### Conflict of interest statement

The authors declare that the research was conducted in the absence of any commercial or financial relationships that could be construed as a potential conflict of interest.
