# Supplementary material for: Genomic Structural Variations Affecting Virulence During Clonal Expansion of Pseudomonas syringae pv. actinidiae Biovar 3 in Europe
Source: Front Microbiol. 2018 Apr 5;9:656. doi: 10.3389/fmicb.2018.00656 (PMC5895724; doi:10.3389/fmicb.2018.00656)
Supplement: Supplementary file 1 [file DataSheet1.docx]

Supplementary Material

**Genomic structural variations affecting virulence during clonal expansion of *Pseudomonas syringae* pv. *actinidiae* biovar 3 in Europe**

*Giuseppe Firrao^1-2^, Emanuela Torelli^1,†^, Cesare Polano^1^, Patrizia Ferrante^3^, Francesca Ferrini^1^, Marta Martini^1^, Simone Marcelletti^3^, Marco Scortichini^3^, Paolo Ermacora^1,^**

*^1^ Department of Agricultural, Food, Environmental and Animal Sciences, University of Udine, Udine, Italy*

*^2^ Istituto Nazionale Biostrutture e Biosistemi, Rome, Italy*

*^3^ Council for Agricultural Research and Analysis of Agricultural Economics (CREA), Research Centre for Olive, Fruit Trees and Citrus, Rome, Italy.*

** Correspondance:*

*Dr. Paolo Ermacora*

[*paolo.ermacora@uniud.it*](mailto:paolo.ermacora@uniud.it)

*Prof. Giuseppe Firrao*

[*giuseppe.firrao@uniud.it*](mailto:giuseppe.firrao@uniud.it)*,*

*Prof. Marco Scortichini*

[*marco.scortichini@crea.gov.it*](mailto:marco.scortichini@crea.gov.it)

† *Present Address*:

*School of Computing Science, ICOS group, Centre for Bacterial Cell Biology. Newcastle University, Newcastle upon Tyne, UK.*

**Running title**: Genomic structural variation in Psa isolates

**Keywords: bacterial canker,** **genomic diversity, hypersensitivity reaction (HR), Illumina technology, single molecule real-time (SMRT) sequencing**

**Supplementary Tables**

**Table S1:** PCR primers used in this work. Expected PCR products were fX1/rX2 (933 bp); fX1/rX4 (686 bp); fX3/rX4 (739 bp).

| Primer name | Primer sequence 5’-3’ |
| --- | --- |
| fX1 | TAGCCACGGTTTTCTTTGCT |
| rX2 | GACGTTTTACCCCATGCACT |
| fX3 | TTCACGGCCAAGAACAACTG |
| rX4 | CCGCTGACTCGTCTTCTCTC |
|  |  |
|  |  |

**Table S2:** Structural variation events distinguishing the chromosomes of strains CRAFRU 14.08 and CRAFRU 12.29.

|  | **Position** | | | |
| --- | --- | --- | --- | --- |
| **Event** | **12.29_left** | **12.29_right** | **14.08_left** | **14.08_right** |
| ISPsy31 insertion | 1.474.713 | 1.476.386 | 1.474.713 | 1.474.714 |
| Chromosomal inversion | 1.852.631 | 5.490.147 | 5.490.627 | 1.850.961 |
| ISPsy37 insertion | 2.118.457 | 2.118.458 | 5.224.801 | 5.223.546 |
| SNP | 3.409.171 | / | 3.932.833 | / |
| VNTR | 4.554.472 | 4.554.473 | 2.787.533 | 2.786.633 |
| SNP | 4.604.846 | / | 2.736.260 | / |

**Table S3:** Structural variation events distinguishing the chromosomes of strains ICMP 18884 and CRAFRU 12.29.

|  | **Position** | | | |
| --- | --- | --- | --- | --- |
| **Event** | **12.29_left** | **12.29_right** | **18708_left** | **18708_right** |
| reverse transcriptase/maturase insertion | 1023375 | 1025252 | 1023375 | / |
| reverse transcriptase/maturase insertion | 5708293 | / | 5715260 | 5717133 |
| Transposase insertion | 3694419 | / | 3287490 | 3288700 |
| chromosomal inversion | 3380078 | 3697838 | 3284077 | 3603006 |
| IS631 transposase insertion | 6513331 | / | 6522179 | 6523356 |
| VNTR | 4554392 |  | 4459558 | 4460460 |
| VNTR | 4824720 |  | 4730787 | 4730844 |

**Table S4:** Gene finding and annotation in filtered assemblies of reads not mapping on the CRAFRU 12.29 chromosome.

| Strain | Contig | Pos. Start | Pos. End | Strand | Annotation |
| --- | --- | --- | --- | --- | --- |
| CRAFRU 14.25 | 3 | 1694 | 1236 | - | hypothetical protein |
| CRAFRU 14.25 | 3 | 1951 | 1691 | - | Phage single stranded DNA synthesis |
| CRAFRU 14.25 | 3 | 3543 | 1948 | - | Phage DNA replication protein |
| CRAFRU 14.25 | 3 | 4539 | 3553 | - | Phage minor capsid protein - DNA pilot protein |
| CRAFRU 14.25 | 3 | 5120 | 4548 | - | Phage major spike protein |
| CRAFRU 14.25 | 3 | 5332 | 5186 | - | Phage major capsid protein |
| CRAFRU 12.54 | 1 | 535 | 275 | - | Phage single stranded DNA synthesis |
| CRAFRU 12.54 | 1 | 2127 | 532 | - | Phage DNA replication protein |
| CRAFRU 12.54 | 1 | 3123 | 2137 | - | Phage minor capsid protein - DNA pilot protein |
| CRAFRU 12.54 | 1 | 3704 | 3132 | - | Phage major spike protein |
| CRAFRU 12.54 | 31 | 943 | 29 | - | Phage major capsid protein |
| CRAFRU 12.54 | 43 | 487 | 642 | + | Error-prone, lesion bypass DNA polymerase V (UmuC) |
| CRAFRU 12.54 | 46 | 620 | 453 | - | hypothetical protein |
| CRAFRU 12.54 | 48 | 410 | 120 | - | Lyzozyme M1 (1,4-beta-N-acetylmuramidase) |
| CRAFRU 12.54 | 49 | 441 | 298 | - | hypothetical protein |
| CRAFRU 12.54 | 49 | 440 | 553 | + | hypothetical protein |
| CRAFRU 12.29 | 2 | 31 | 465 | + | Phage major capsid protein |
| CRAFRU 12.29 | 2 | 531 | 1103 | + | Phage major spike protein |
| CRAFRU 12.29 | 2 | 1112 | 2098 | + | Phage minor capsid protein - DNA pilot protein |
| CRAFRU 12.29 | 2 | 2108 | 3703 | + | Phage DNA replication protein |
| CRAFRU 12.29 | 2 | 3700 | 3960 | + | Phage single stranded DNA synthesis |
| CRAFRU 12.29 | 2 | 3957 | 4415 | + | hypothetical protein |
| CRAFRU 14.21 | 1 | 7 | 654 | + | Phage major capsid protein |
| CRAFRU 14.21 | 1 | 720 | 1292 | + | Phage major spike protein |
| CRAFRU 14.21 | 1 | 1301 | 2287 | + | Phage minor capsid protein - DNA pilot protein |
| CRAFRU 14.21 | 16 | 620 | 453 | - | hypothetical protein |
| CRAFRU 14.21 | 3 | 1 | 579 | + | Phage DNA replication protein |
| CRAFRU 14.21 | 3 | 576 | 836 | + | Phage single stranded DNA synthesis |
| CRAFRU 14.21 | 40 | 192 | 79 | - | hypothetical protein |
| CRAFRU 14.21 | 40 | 191 | 334 | + | hypothetical protein |
| CRAFRU 14.08 | 1 | 535 | 275 | - | Phage single stranded DNA synthesis |
| CRAFRU 14.08 | 1 | 2127 | 532 | - | Phage DNA replication protein |
| CRAFRU 14.08 | 1 | 3123 | 2137 | - | Phage minor capsid protein - DNA pilot protein |
| CRAFRU 14.08 | 1 | 3704 | 3132 | - | Phage major spike protein |
| CRAFRU 14.08 | 1 | 4684 | 3770 | - | Phage major capsid protein |
| CRAFRU 14.08 | 14 | 750 | 127 | - | Lyzozyme M1 (1,4-beta-N-acetylmuramidase) |
| CRAFRU 14.08 | 22 | 169 | 56 | - | hypothetical protein |
| CRAFRU 14.08 | 22 | 168 | 311 | + | hypothetical protein |
| CRAFRU 14.08 | 29 | 620 | 453 | - | hypothetical protein |
| CRAFRU 14.08 | 63 | 191 | 316 | + | hypothetical protein |
| CRAFRU 14.08 | 73 | 168 | 37 | - | hypothetical protein |
| CRAFRU 13.27 | 4 | 1399 | 941 | - | hypothetical protein |
| CRAFRU 13.27 | 4 | 1656 | 1396 | - | Phage single stranded DNA synthesis |
| CRAFRU 13.27 | 4 | 3248 | 1653 | - | Phage DNA replication protein |
| CRAFRU 13.27 | 4 | 4244 | 3258 | - | Phage minor capsid protein - DNA pilot protein |
| CRAFRU 13.27 | 4 | 4825 | 4253 | - | Phage major spike protein |
| CRAFRU 13.27 | 4 | 5358 | 4891 | - | Phage major capsid protein |
| CRAFRU 14.10 | 4 | 905 | 447 | - | Phage external scaffolding protein #Protein D |
| CRAFRU 14.10 | 4 | 1162 | 902 | - | Phage single stranded DNA synthesis |
| CRAFRU 14.10 | 4 | 2754 | 1159 | - | Phage DNA replication protein |
| CRAFRU 14.10 | 4 | 3750 | 2764 | - | Phage minor capsid protein - DNA pilot protein |
| CRAFRU 14.10 | 4 | 4331 | 3759 | - | Phage major spike protein |
| CRAFRU 14.10 | 4 | 5311 | 4397 | - | Phage major capsid protein |
| CRAFRU 12.50 | 14 | 577 | 317 | - | Phage single stranded DNA synthesis |
| CRAFRU 12.50 | 14 | 1152 | 574 | - | Phage DNA replication protein |
| CRAFRU 12.50 | 18 | 1 | 684 | + | Phage minor capsid protein - DNA pilot protein |
| CRAFRU 12.50 | 36 | 60 | 227 | + | hypothetical protein |
| CRAFRU 12.50 | 39 | 320 | 30 | - | Lyzozyme M1 (1,4-beta-N-acetylmuramidase) |
| CRAFRU 12.50 | 8 | 7 | 654 | + | Phage major capsid protein |
| CRAFRU 12.50 | 8 | 720 | 1292 | + | Phage major spike protein |
| CRAFRU 12.64 | 1 | 1749 | 1066 | - | Phage minor capsid protein - DNA pilot protein |
| CRAFRU 12.64 | 10 | 19 | 549 | + | Phage DNA replication protein |
| CRAFRU 12.64 | 14 | 576 | 433 | - | hypothetical protein |
| CRAFRU 12.64 | 14 | 575 | 688 | + | hypothetical protein |
| CRAFRU 12.64 | 17 | 620 | 453 | - | hypothetical protein |
| CRAFRU 12.64 | 4 | 79 | 651 | + | Phage major spike protein |
| CRAFRU 12.64 | 43 | 143 | 18 | - | hypothetical protein |
| CRAFRU 10.29 | 1 | 102 | 449 | + | Phage minor capsid protein - DNA pilot protein |
| CRAFRU 10.29 | 10 | 30 | 224 | + | Phage major spike protein |
| CRAFRU 10.29 | 121 | 198 | 55 | - | hypothetical protein |
| CRAFRU 10.29 | 16 | 535 | 275 | - | Phage single stranded DNA synthesis |
| CRAFRU 10.29 | 18 | 564 | 397 | - | Phage external scaffolding protein #Protein D |
| CRAFRU 10.29 | 25 | 422 | 132 | - | Lyzozyme M1 (1,4-beta-N-acetylmuramidase) |
| CRAFRU 10.29 | 6 | 7 | 654 | + | Phage major capsid protein |
| CRAFRU 10.29 | 89 | 138 | 269 | + | hypothetical protein |

## Supplementary Figures


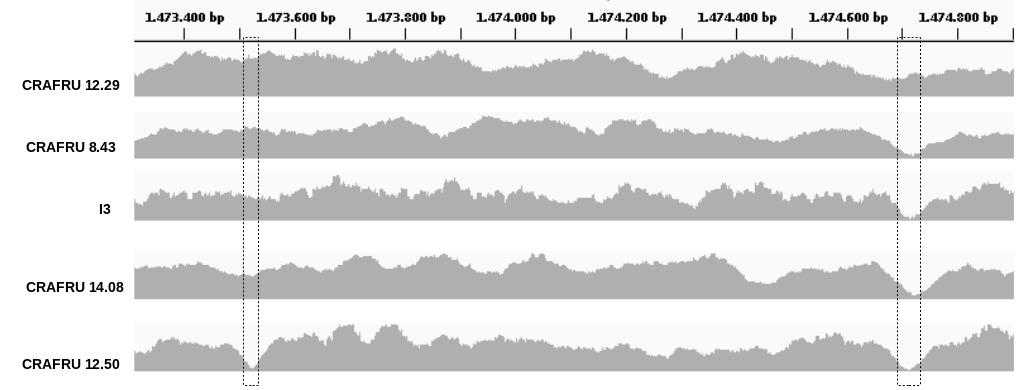


**Figure S1:** Coverage histograms of positions 1,473,400 - 1,474,800 (CRAFRU 12.29 numbering) with Illumina reads from strain CRAFRU 12.29, CRAFRU 8.43, I3, CRAFRU 14.08, and CRAFRU 12.50. The regions of transposon insertion are marked with a dotted box. The box on the left show a coverage gap in strain CRAFRU 12.50 due to the insertion of transposon ISPsy36 at position 1,473,520. In the box on the right all strains but CRAFRU 12.29 show a coverage gap since CRAFRU 12.29, that is used as a reference, has a copy of ISPsy31 inserted at position 1,474,793.


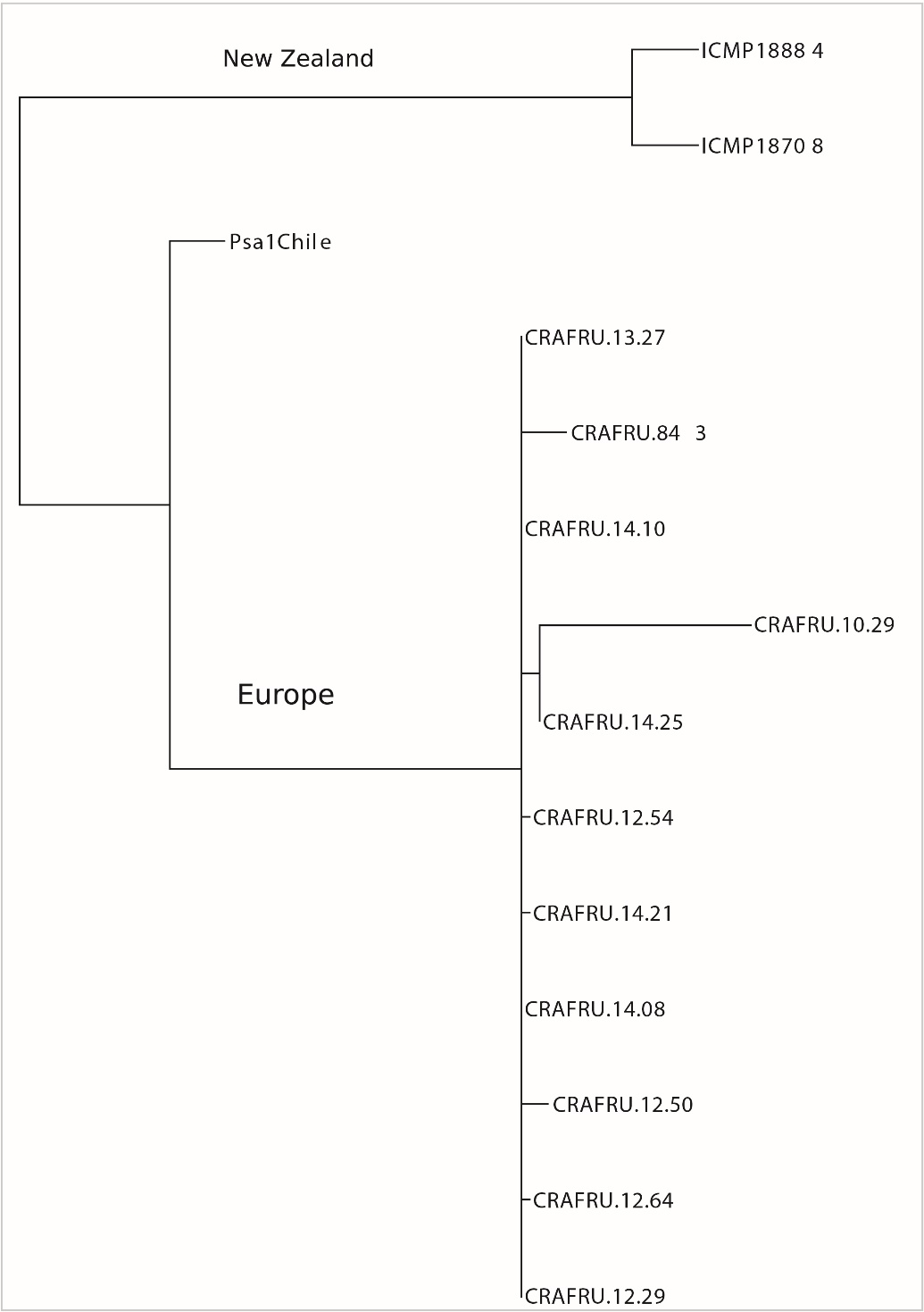


**Figure S2.** Tree based on distance among genomes of the strains used in this work (and one additional strain included for reference, Psa1Chile, unpublished result) calculated from SNPs.


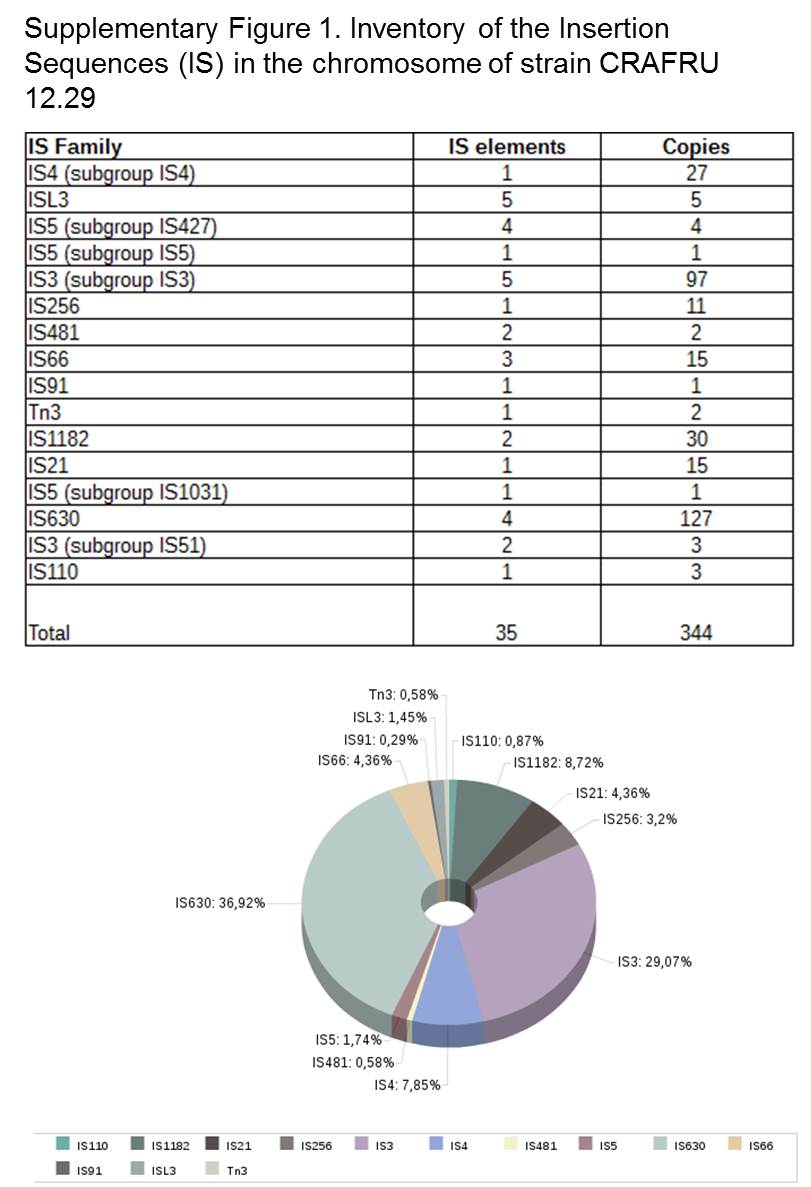


**Figure S3:** Inventory of the Insertion Sequences (IS) in the chromosome of strain CRAFRU 12.29.
